# The Wildlife Malaria Research network (WIMANET): Meeting report on the 1st WIMANET workshop

**DOI:** 10.1016/j.ijppaw.2024.100989

**Published:** 2024-09-12

**Authors:** Rafael Gutiérrez-López, Martina Ferraguti, Kasun H. Bodawatta, Carolina R.F. Chagas, Nayden Chakarov, Mélanie Duc, Tamara Emmenegger, Luz García-Longoria, Ricardo J. Lopes, Josué Martínez-de la Puente, Swen C. Renner, Diego Santiago-Alarcon, Ravinder N.M. Sehgal, Daliborka Stankovic, Alfonso Marzal, Jenny C. Dunn

**Affiliations:** a National Center of Microbiology, Carlos III Health institute. Ctra de Pozuelo km 2, Majadahonda, Madrid, Spain; b CIBER de Enfermedades Infecciosas (CIBERINFEC), Madrid, Spain; c Doñana Biological Station (EBD), CSIC, Department of Conservation Biology and Global Change, C/ Américo Vespucio, 26, 41092, Seville, Spain; d CIBER de Epidemiología y Salud Pública (CIBERESP), Madrid, Spain; e Section for Molecular Ecology and Evolution, Globe Institute, University of Copenhagen, Copenhagen, Denmark; f Nature Research Centre, Akademijos g. 2, 08412, Vilnius, Lithuania; g Department of Animal Behaviour, Bielefeld University, Konsequenz 45, 33615, Bielefeld, Germany; h JICE, Joint Institute for Individualisation in a Changing Environment, Bielefeld University, Germany; i Museum Luzern, Department of Zoology, Kasernenplatz 6, 6003, Lucerne, Switzerland; j Department of Anatomy, Cell Biology and Zoology. Avenida de Elvas, edificio Margarita Salas CP 06006 Universidad de Extremadura, Badajoz, Spain; k cE3c, Center for Ecology, Evolution and Environmental Change & CHANGE, Global Change and Sustainability Institute, Departamento de Biologia Animal, Faculdade de Ciências, Universidade de Lisboa, 1749-016, Lisboa, Portugal; l MHNC-UP, Natural History and Science Museum of the University of Porto, Porto, Portugal; m Natural History Museum Vienna, Austria; n Department of Integrative Biology, University of South Florida, Tampa, FL, USA; o Department of Biology, San Francisco State University, California, San Francisco, USA; p University in Belgrade – Institute for Multidisciplinary Research, Kneza Višeslava 1, 11030, Belgrade, Serbia; q Wildlife Research Group, National University of San Martin, Tarapoto, Peru; r School of Life Sciences, Keele University, Newcastle-under-Lyme, Staffordshire, ST5 5BG, UK

**Keywords:** COST-Action, Networking, Capacity building, Vertebrate-hosts, Insect-vectors, Haemosporidians

## Abstract

The Wildlife Malaria Network (WIMANET) is a groundbreaking multinational collaboration focused on studying vector-borne haemosporidian parasites in wildlife. Unlike human malaria, wildlife malaria is found on all continents except Antarctica, with parasites being transmitted by a range of vectors. The complexity and diversity of these parasites makes it necessary to have an interdisciplinary approach to understand and mitigate their impacts. Established in 2023 within the framework of COST-Action (European Cooperation in Science and Technology), WIMANET unites researchers from diverse scientific backgrounds to tackle critical questions about wildlife malaria on a global scale. This meeting report summarises the activities and plans resulting from the 1^st^ meeting of WIMANET's six working groups, spanning the genetic and morphological identification of parasites to understanding the drivers of host-parasite-vector associations from individual to community levels. WIMANET's collaborative efforts aim to fill the knowledge gaps and foster large-scale research initiatives transcending local and regional boundaries.

## Highlights

• WIMANET is a global collaborative network working on wildlife malaria parasites.
• WIMANET consists of six working groups (WGs), each focused on different aspects of wildlife malaria research.
• WIMANET focus on equipping early-career researchers with the tools and skills needed to study wildlife malaria.

## Graphical abstract

**Figure:**
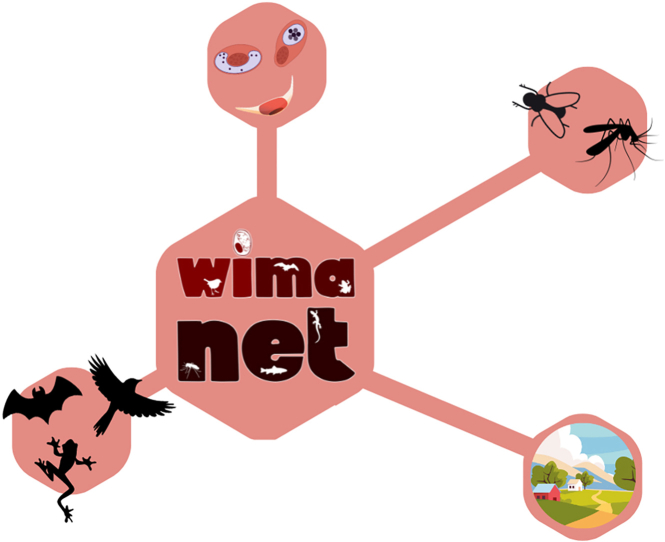

## 1 Introducing WIMANET: A global network for advancing wildlife malaria research

The **Wi**ldlife **Ma**laria **Net**work (WIMANET; EU COST Action CA22108), is a multinational network of researchers established under the framework of EU COST (European Cooperation in Science and Technology). The primary aim of WIMANET is to consolidate resources, expertise, and data from research groups studying wildlife malaria parasites to address key questions on a global scale. Currently, vector-borne diseases pose an increasing concern to the health of humans, livestock and wildlife (Chala and Hamde, 2021). Among these, wildlife malaria parasites, including *Plasmodium* spp. and other vector-borne protozoa, are remarkably diverse in wildlife, encompassing non-human mammals, birds, reptiles, amphibians and fish (Gutiérrez-López et al., 2023; Santiago-Alarcon and Marzal, 2020). These parasites are found on all continents except in Antarctica and are transmitted by a diversity of insect vectors (Valkiūnas, 2005). Their study spans a wide range of disciplines, including molecular genetics, parasitology, evolutionary biology, ecology, epidemiology, taxonomy, entomology, and veterinary science, among many others, using from traditional methods to modern bioinformatic tools (Valkiūnas and Atkinson, 2020).

**Fig. 1:**
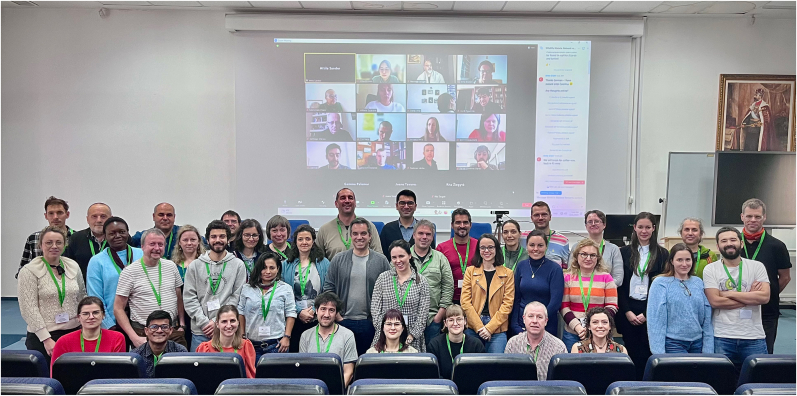
A total of 84 people participated at the 1^st^ hybrid WIMANET workshop, celebrated at the University of Agricultural Sciences and Veterinary Medicine, Cluj-Napoca, Romania from February 20th to 23rd, 2024.

To foster interdisciplinary knowledge sharing and enable multinational collaborations, a Wildlife Malaria Network was initiated in 2023 within the COST framework. During the first WIMANET workshop, celebrated in a hybrid format (mixed in-person and online) at the University of Agricultural Sciences and Veterinary Medicine, Cluj-Napoca, Romania from February 20th to 23rd, 2024 (Fig. 1), six specialized working groups (WGs) presented and brainstormed ideas covering gaps in knowledge, experimental designs, protocols, and existing databases of wildlife malaria parasites, hosts, and vectors.

The following provides an overview of the six WGs and their missions:

### 1.1 WG1: Molecular markers and genomics

WG1 focuses on identifying key molecular markers for genomics and transcriptomic analyses in wildlife malaria research. The group is organizing a comprehensive review on genomics and transcriptomic analysis methods, including RNA-seq and single-cell sequencing, with an emphasis on standardizing protocols and providing training. The immediate goal is to compile and review available methods for intracellular parasites and to discuss the limitations and perspectives of cutting-edge omics techniques. WG1 also plans to organize a training school focusing on bioinformatic techniques for working with genomic datasets, potentially online.

### 1.2 WG2: Species identification and phylogenetic relationships

WG2 aims to integrate molecular markers and morphological data to assign parasite lineages to species and elucidate phylogenetic relationships. The group introduced three new initiatives to enhance species identification skills and methodologies, including the presentation of 'Monthly Cases' for identification, and an interactive identification key software for haemosporidian parasites, initially focusing on avian haemosporidians as these are currently better characterized than haemosporidians from other host taxa. Additionally, WG2 plans to compile sample collection protocols and publish review papers on parasite identification methods.

### 1.3 WG3: Vector transmission in wildlife

WG3 investigates factors influencing the transmission of wildlife malaria parasites by insect vectors. The group initially proposes to write review papers on wildlife malaria vectors, categorized by vertebrate groups (birds, reptiles, amphibians, and mammals). They also plan to establish standardized protocols for wildlife malaria research. A significant goal is to develop a network interaction map of vectors and their parasites, requiring data from various countries. WG3 encourages members to share sampling and processing protocols for the study of the interactions between wildlife malaria parasites, vertebrate hosts and insect vectors.

### 1.4 WG4: Anthropogenic impacts on host haematology

WG4 explores the impact of anthropogenic activities and wildlife malaria on host haematology. The group discussed using machine learning (ML) to automate cell counts from blood smears. The objective is to develop an ML-based tool for detecting infected erythrocytes and other blood cells, providing detailed analyses and summary spreadsheets. WG4 also aims to create a Slack channel for discussing haematology and parasite determination photos and to establish a metadata database of currently available haematological data that will be available to contribute to large-scale analyses.

### 1.5 WG5: Spatiotemporal variation in host-parasite communities

WG5 focuses on identifying the biotic and abiotic drivers influencing host-malaria associations across vertebrate clades. The group emphasized community-level studies of hosts and their malaria parasites, with plans to expand beyond birds to include other vertebrate taxa such as reptiles, amphibians, and mammals. WG5 has identified several research questions, including the influence of biotic and abiotic factors on host-parasite networks and the impact of land use on these networks. It plans to initially define the scale and size of the community, before gathering datasets from various sources and generating standardized sample collection protocols on multi-host-parasite communities.

### 1.6 WG6: Dissemination and public engagement

WG6 is responsible for coordinating, disseminating and publicizing WIMANET's findings. This group is involved in several initiatives, including the organization of summer schools, training videos, newsletters, and social media engagement, currently active on platforms such as Instagram (wimanetscience) and X (@WIMANETscience). Also, WG6 plans to develop brochures in multiple languages for dissemination at different conferences. The group also proposes preparing meeting reports for publication in a scientific journal to further promote WIMANET's activities.

A recurring theme from all Working Group discussions was the need for a book to compile standardized protocols that will enable the generation of comparable data from all groups working on wildlife haemosporidians. Therefore, in the concluding session, it was planned for Working Groups to combine efforts to produce a Handbook of Field Techniques for working with wildlife haemosporidian. This book will focus on field techniques rather than laboratory techniques, encompassing the full range of wildlife haemosporidian hosts and vectors, including collecting and storing samples for a range of downstream applications, and techniques for basic analyses that can be conducted in the field such as initial microscopy screening.

## 2 What's next? Join WIMANET

**Fig. 2:**
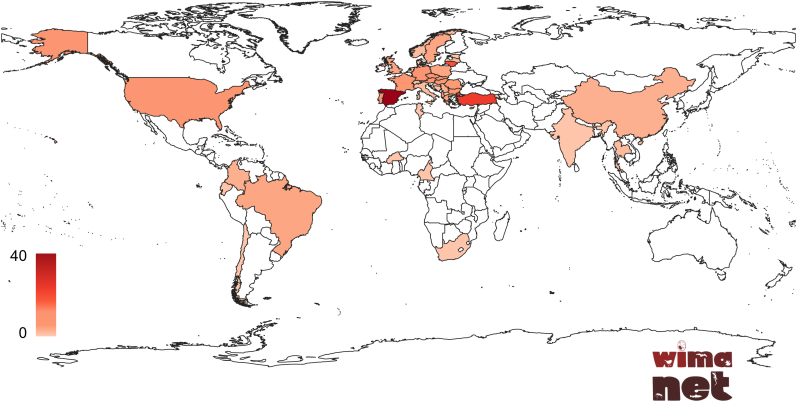
Countries of origin of the members participating in WIMANET. The colour intensity bar indicates the number of members per country. (For interpretation of the references to colour in this figure legend, the reader is referred to the Web version of this article.)

One of WIMANET's primary objectives is to empower and equip young and early-career researchers with the essential skills required for wildlife parasitology research. WIMANET is more than a research network; it is a collaborative platform dedicated to addressing the critical issues of wildlife malaria on a global scale. In line with this commitment, the WIMANET will be organizing a series of short scientific stays and training schools aimed at teaching young researchers the methods used to study vector-borne parasites. By joining WIMANET, you will contribute to pioneering research and become part of a dynamic community of scientists committed to making a significant impact. Visit the official website https://wimanet-science.github.io/web/and the COST Action website (https://www.cost.eu/actions/CA22108/) to apply through the e-COST platform to become a member. Stay updated by following WIMANET on social media (@WIMANETscience) and subscribing to our newsletter. This Action COST has currently more than 200 leading scientists from 41 countries, and is continuously growing (Fig. 2). Currently, 48% of members are female, 56% are younger researchers (under 40 years old), and 50% are from Inclusiveness Target Country (ITC) countries. Together, WIMANET can advance the understanding and management of wildlife malaria, fostering a healthier ecosystem.

## Funding

This work was conducted within the framework of the WIMANET-COST Action CA22108.

## Data availability

Not applicable.

## Ethics

This work involved no human subjects.

## CRediT authorship contribution statement

**Rafael Gutiérrez-López:** Writing – review & editing, Writing – original draft, Supervision, Investigation, Data curation, Conceptualization. **Martina Ferraguti:** Writing – review & editing, Supervision, Methodology, Investigation, Data curation, Conceptualization. **Kasun H. Bodawatta:** Writing – review & editing. **Carolina R.F. Chagas:** Writing – review & editing. **Nayden Chakarov:** Writing – review & editing. **Mélanie Duc:** Writing – review & editing, Conceptualization. **Tamara Emmenegger:** Writing – review & editing. **Luz García-Longoria:** Writing – review & editing, Conceptualization. **Ricardo J. Lopes:** Writing – review & editing. **Josué Martínez-de la Puente:** Writing – review & editing. **Swen C. Renner:** Writing – review & editing. **Diego Santiago-Alarcon:** Writing – review & editing. **Ravinder N.M. Sehgal:** Writing – review & editing. **Daliborka Stankovic:** Writing – review & editing. **Alfonso Marzal:** Writing – review & editing. **Jenny C. Dunn:** Writing – review & editing, Supervision, Funding acquisition.

## Declaration of competing interest

The authors declare that they have no known competing financial interests or personal relationships that could have appeared to influence the work reported in this paper.
