# Corrigendum to “The Wildlife Malaria Research network (WIMANET): Meeting report on the 1st WIMANET workshop” [Int. J. Parasitol: Parasites and Wildlife 25 (2024) 100989]

**DOI:** 10.1016/j.ijppaw.2025.101059

**Published:** 2025-07-15

**Authors:** Rafael Gutiérrez-López, Martina Ferraguti, Kasun H. Bodawatta, Carolina R.F. Chagas, Nayden Chakarov, Mélanie Duc, Tamara Emmenegger, Luz García-Longoria, Ricardo J. Lopes, Josué Martínez-de la Puente, Swen C. Renner, Diego Santiago-Alarcon, Ravinder N.M. Sehgal, Daliborka Stankovic, Alfonso Marzal, Jenny C. Dunn

**Affiliations:** a National Center of Microbiology, Carlos III Health Institute. Ctra de Pozuelo km 2, Majadahonda, Madrid, Spain; b CIBER de Enfermedades Infecciosas (CIBERINFEC), Madrid, Spain; c Doñana Biological Station (EBD), CSIC, Department of Conservation Biology and Global Change, C/ Américo Vespucio, 26, 41092, Seville, Spain; d CIBER de Epidemiología y Salud Pública (CIBERESP), Madrid, Spain; e Section for Molecular Ecology and Evolution, Globe Institute, University of Copenhagen, Copenhagen, Denmark; f Nature Research Centre, Akademijos g. 2, 08412, Vilnius, Lithuania; g Department of Animal Behaviour, Bielefeld University, Konsequenz 45, 33615, Bielefeld, Germany; h JICE, Joint Institute for Individualisation in a Changing Environment, Bielefeld University, Germany; i Museum Luzern, Department of Zoology, Kasernenplatz 6, 6003, Lucerne, Switzerland; j Department of Anatomy, Cell Biology and Zoology. Avenida de Elvas, Edificio Margarita Salas CP 06006 Universidad de Extremadura, Badajoz, Spain; k CE3c, Center for Ecology, Evolution and Environmental Change & CHANGE, Global Change and Sustainability Institute, Departamento de Biologia Animal, Faculdade de Ciências, Universidade de Lisboa, 1749-016, Lisboa, Portugal; l MHNC-UP, Natural History and Science Museum of the University of Porto, Porto, Portugal; m Natural History Museum Vienna, Austria; n Department of Integrative Biology, University of South Florida, Tampa, FL, USA; o Department of Biology, San Francisco State University, San Francisco, CA, USA; p University in Belgrade – Institute for Multidisciplinary Research, Kneza Višeslava 1, 11030, Belgrade, Serbia; q Wildlife Research Group, National University of San Martin, Tarapoto, Peru; r School of Life Sciences, Keele University, Newcastle-under-Lyme, Staffordshire, ST5 5BG, UK

The authors regret < This article is based upon work from 10.13039/501100000921COST Action Wildlife Malaria 10.13039/100031212Network (WIMANET), CA22108, supported by 10.13039/501100000921COST (10.13039/501100000921European Cooperation in Science and Technology).

The authors would like to apologise for any inconvenience caused.

